# Effectiveness of Radioiodine Treatment for Toxic Nodular Goiter

**DOI:** 10.4274/mirt.48378

**Published:** 2015-11-02

**Authors:** Hatice Şakı, Arzu Cengiz, Yakup Yürekli

**Affiliations:** 1 Adnan Menderes University Faculty of Medicine, Department of Nuclear Medicine, Aydın, Turkey

**Keywords:** Hyperthyroidism, Nodular goiter, iodine 131

## Abstract

**Objective:**

The aim of this retrospective study is to evaluate the treatment outcomes in patients with toxic nodular goiter (TNG) that received radioiodine treatment (RAIT) and to determine the influence of age, gender, nodule size, I-131 dose, underlying etiology and antithyroid drugs on the outcomes of RAIT.

**Methods:**

Two hundred thirty three patients (mean 64±10 years old) with TNG that received RAIT were included in the study. Treatment success was analyzed according to demographic (age and gender) and clinical data (thyroid function tests before and after RAIT, thyroid sonography and scintigraphy, I-131 dose, antithyroid drugs). A fixed dose of 555 MBq was administered to patients with nodules smaller than 2 cm in diameter and of 740 MBq to patients with nodules larger than 2 cm. Hyperthyroidism treatment success was defined as achieving hypothyroidism or euthyroidism six months after RAIT.

**Results:**

In our study, the cure rate was 93.9% six months after RAIT. Hypothyroidism was observed in 74 (31.7%) patients, and euthyroidism was achieved in 145 (62.2%) patients while 14 (6%) patients remained in hyperthyroid state. Age and gender did not affect treatment outcomes. No correlation was found between underlying etiology or antithyroid drugs and therapeutic effectiveness. The effectiveness of RAIT was better in patients with nodules smaller than 2 cm.

**Conclusion:**

We observed that high cure rates were obtained in patients with TNG with 555 MBq and 740 MBq doses of I-131. While nodule diameter and RAI dose are important factors for treatment efficacy; age, gender, underlying etiology and antithyroid drugs do not affect the outcome of RAIT.

## Abstract (tr)

**Amaç:**

Bu retrospektif çalışmada amaç, radyoaktif iyot tedavisi (RAIT) alan toksik nodüler guatr (TNG) hastalarında tedavi sonuçlarını değerlendirerek hasta yaşı, cinsiyet, nodül çapı, kullanılan I-131 dozu, altta yatan etiyoloji ve antitiroid ilaç kullanımı gibi faktörlerin tedavi sonuçları üzerine etkisini değerlendirmektir.

**Yöntem:**

TNG tanısıyla RAIT alan 233 hasta (64±10 yaş) çalışmaya dahil edildi. Hastalara ait demografik (yaş ve cinsiyet) ve klinik veriler (tedavi öncesi ve sonrası tiroid fonksiyon testleri, tiroid sintigrafisi ve ultrasonografi sonuçları, I-131 dozu ve antitiroid ilaç kullanımı) değerlendirildi. Nodül çapı 2 cm’den küçük olan hastalara 555 MBq ve nodül çapı 2 cm’den büyük olanlara 740 MBq olacak şekilde sabit doz RAIT verildi. Altıncı ayda hipotiroidi ve ötiroidi gelişen hastalar kür olarak kabul edildi.

**Bulgular:**

Çalışmamızda RAIT verildikten sonra altıncı ayda kür oranı %93,9 idi ve hastaların 74’ünde (%31,7) hipotiroidi, 145’inde (%62,2) ötiroidi ve 14’ünde (%6) hipertiroidi olduğu görüldü. Yaş ve cinsiyetin tedavi etkinliğini değiştirmediği görüldü. Altta yatan etiyoloji ve antitiroid kullanımı ile tedavi etkinliği arasında anlamlı bir ilişki saptanmadı. Nodül çapı 2 cm’den küçük olan hastalarda RAIT’in daha başarılı olduğu görüldü.

**Sonuç:**

TNG hastalarında 555 MBq ve 740 MBq I-131 dozları ile yüksek kür oranları sağladık. Nodül çapı ve I-131 dozu tedavi etkinliği için önemli faktörler iken yaş, cinsiyet, altta yatan etiyoloji ve antitiroid ilaç kullanımı RAIT sonuçlarında etkili olmamaktadır.

## INTRODUCTION

Hyperthyroidism is a pathological syndrome in which tissues are exposed to excessively increased amount of thyroid hormones. The most common reason for hyperthyroidism is Graves’ disease (GD). Toxic nodular goiter (TNG) is a clinical situation that includes toxic multinodular goiter (TMNG) and toxic adenoma (TA), and is the second most common reason of hyperthyroidism (1). Antithyroid drugs (ATD), radioactive iodine (RAI-131) therapy and surgery are the most common treatment methods for hyperthyroidism. Typically, ATD are preferred as the initial treatment, however, the treatment generally does not provide long-term control and result in high recurrence rates after discontinuance, and has certain drug-related adverse effects. In surgery, the treatment rate is high, but serious complications such as recurrent laryngeal nerve injury and hypoparathyroidism may be seen. That is why, RAI is used commonly due to its relatively low cost, reasonable half-life of 8 days, and favorable clinical outcome (2,3).

This study which focused on patients who had received RAI-131 therapy (RAIT) after being referred to our clinic with a TNG diagnosis, aimed to evaluate the therapeutic efficacy of I-131 in TNG patients by studying factors such as initial nodule diameter, age, gender, antithyroid drug use, the underlying etiology and total dose of RAI-131.

## MATERIALS AND METHODS

All patients who were referred to our department for RAIT and received RAIT with a diagnosis of TNG were retrospectively evaluated. Two hundred thirty-three patients who attended regular follow-up appointments during a 1-year period after receiving RAIT and who had complete clinical and laboratory data in the post-treatment follow-up period were included. Five patients who received a second dose of I-131 therapy during their follow-up due to on-going hyperthyroidism were excluded from the study.

The study data were extracted from the I-131 treatment and follow-up forms. Demographic data such as age and gender, thyroid scintigraphy and ultrasonography (USG) imaging findings, thyroid function tests performed before treatment and in post-treatment follow-up, the administered I-131 dose and pre-treatment ATD use were evaluated. Treatment success was evaluated based on thyroid function tests performed at the post-treatment 6th and 12th-month. Patients who developed euthyroidism and hypothyroidism in the 6th month were accepted as cured.

Patients with laboratory findings of hyperthyroidism (free thyroxine (sT4) high or normal, thyroid stimulating hormone (TSH) low) and whose clinical values were found to be compatible with hyperthyroidism were grouped as TA and TMNG according to physical examination, thyroid USG and/or scintigraphy.

Two different fixed doses were administered according to the initial nodule diameter and used ATD dose, patient’s age and size of thyroid gland. Seven hundred forty MBq was administered to older patients with large thyroid glands, and if the largest nodule diameter was >2 cm on USG, while 555 MBq was administered to younger patients with normal-sized or slightly enlarged thyroid gland and a nodule diameter of ≤2 cm. RAI uptake was not evaluated. Informed consent was obtained from all patients before RAI treatment. Patients were informed about complying with the diet list starting 10 days before treatment, in order to ensure low iodine intake in their diets. Iodine-containing drugs and preparations were discontinued before treatment. In patients who used ATD prior to treatment, the ATD that has been discontinued one week before treatment was started again one week after treatment. Following treatment, patients were evaluated at 1, 3, 6, and 12 months for thyroid function tests, clinical symptoms physical examination (PM) and ATD dose. ATD dose was reduced in those patients whose clinical condition improved and whose sT4 values decreased and/or TSH levels increased as suggested by thyroid function tests. ATD treatment was discontinued if either subclinical (sT4 normal, TSH high), or overt hypothyroidism (sT4 low, TSH high), or euthyroidism (sT4 and TSH normal) was achieved. Euthyroidism or hypothyroidism (subclinical/overt hypothyroidism) as suggested by the thyroid function tests, after discontinuing ATD, was accepted as treatment success.

### Statistical Analysis

Pearson, Yates or Fisher Exact Chi-square tests were used for analysis depending on the theoretical frequency value, while average ± standard deviation, frequency and percentage values were used for descriptive statistics. A value of p<0.05 was regarded as statistically significant.

## RESULTS

The results of the study showed a cure rate of 93.9%. At the 6-month follow-up examination, 74 out of 233 patients (31.7%) had hypothyroidism, 145 (62.2%) had euthyroidism and 14 (6%) had hyperthyroidism.

The 233 patients included in the study included 163 (70%) females and 70 (30%) males. The cure rate was found to be 95.1% in females and 91.4% in males. There was no statistically significant difference between genders in terms of treatment success (p>0.05).

The patients were between the ages of 28 and 86 years (mean 64±10 years). The treatment results by age group are summarized in Table 1. There was no statistically significant difference between age groups in terms of treatment success (p>0.05).

Fifty-nine patients (25.3%) had a nodule diameter of ≤2 cm, while the nodule diameter was >2 cm in 174 (74.7%). The cure rate in patients with a nodule diameter of ≤2 cm was 100%, while it was 92% in patients with nodules >2 cm. There was a statistically significant difference between groups according to nodule size in terms of treatment success (p<0.05).

In terms of I -131 dose, 81 patients (34.8%) received 555 MBq, and 152 patients (65.2%) 740 MBq. The treatment results of patients according to the administered dose of I-131 are summarized in Table 2. The cure rate was found to be higher in patients who received treatment at 555 MBq dose and the difference was statistically significant (p<0.05).

Thirty-eight patients who received RAIT (16%) were diagnosed with TA and 195 (84%) had TMNG. The cure rate was 94.7% for toxic adenoma, and 93.8% for TMNG. There was no statistically significant difference between these two groups (p>0.05).

Prior to treatment, 194 patients (83.3%) used ATD whereas 39 patients (16.7%) did not. The cure rate was 93.8% in patients who used ATD, and 94.9% in patients who did not. Detailed results of patients according to the use of ATD are shown Table 3. From a treatment response perspective, there was no statistically significant difference between the two groups (p>0.05).

## DISCUSSION

ATD and other methods such as percutaneous ethanol injection and surgery are being used along with RAIT for the treatment of TNG. However, definite and effective treatment can be achieved in TNG only with RAIT and surgery (2,3,4).

Several studies reported that a 6-month period is sufficient to evaluate post-treatment cure rate. For the current study, the cure rate was evaluated according to post-treatment 6-month results, yielding an overall cure rate of 93.9%. While in one study the cure rate at 6 months was found to be 71.4% in a TNG patient (5), there are also studies reporting similar cure rates to our result such as 94% and 92% (6,7). The treatment rates in the current study were compatible with previous studies.

Currently, there is no consensus regarding the appropriate RAIT dose. Some centers give fixed low (185-370 MBq) or high (555-925 MBq) doses, while others recommend that calculated doses be given. A meta-analysis, which evaluated 8 different studies where fixed and calculated doses were compared, reported both methods to be equally successful (8). Allahabadia et al. (5) administered two different fixed doses (185 MBq and 740 MBq) to different patient groups and found a higher cure rate (84.6%) in patients who were given 740 MBq as compared to patients who were given 185 MBq (66.6%), and the rate of hypothyroidism was found to be higher with high doses. Zakavi et al. (9) compared fixed low and high doses (481 MBq vs 832 MBq) with calculated low and high doses (average 388 MBq and 692 MBq) administered to patients with a single hot nodule, and at 6 months the hyperthyroidism rate was found to be lower in patients who have been treated with a high dose (12.5% vs. 31%). In the current study, two different fixed high doses of 555 and 740 MBq were administered according to the initial nodule diameter, patient age and clinical status. Unlike other studies, the cure rate was observed to be higher in patients who received 555 MBq as compared to those who received 740 MBq (100% and 90.8%). This could be explained by the fact that a low dose was given to patients with small nodule diameter and non-severe hyperthyroidism, and that the treatment success was found to be higher in patients with small nodule diameter. Nevertheless, it should be noted that the low doses given in many studies were actually lower than the I-131 dose of 555 MBq used in the current study. The highest I-131 dose allowed in outpatient clinics is limited to 740 MBq, which may have prevented treatment success in high-dose group patients who may need doses higher than the upper limit.

While hypothyroidism has been reported in some studies as a complication, it is a targeted result especially in old patients with cardiac problems in order to achieve long-lasting treatment by triggering hypothyroidism with a single high dose. Post-RAIT hypothyroidism is lower in nodular goiter patients as compared to Graves’ disease. In a study by Metso et al., (10) hypothyroidism incidence at the end of the first year was found to be 24% for Graves’ disease, while it was 4% for TMNG. In a study by Ustun et al., (11) hypothyroidism incidence at the end of 6 months was reported as 20.7% for TA, and 29.6% for TMNG. In the current study, hypothyroidism incidence at 6 months in TNG patients was 36.8% for TA, and 30.8% for TMNG. The higher hypothyroidism rates in the current study as compared to previous studies could be explained by administration of higher I-131 doses than that used in previous studies. While early hypothyroidism risk is associated with the I-131 dose administered, late-onset hypothyroidism is mainly associated with natural disease course. A similar situation may also arise after surgery and ATD use.

In terms of the impact of gender on treatment efficacy, there are studies stating that treatment is less successful in female patients (12), or that there is no correlation between gender and treatment efficacy (6), while some earlier studies reported that RAIT is more successful in females (6,13,14). In the current study, RAIT was more successful in females (95.1% vs. 91.4%), but the difference between genders was not statistically significant.

Although the thyroid gland is assumed to be more resistant to radiation in younger patients, Allahabadia et al. (5) found a lower cure rate in patients below 40 years of age as compared to those above 40 years (68.9% vs. 79.3%). In our patient population, there were only four patients below the age of 40 years, that’s why we selected 50 years as a cut-off. In the current study, the cure rate was highest in patients below the age of 50 years (%100), and was lowest in patients older than 70 years (90.4%) but the difference between age groups was not statistically significant. Knapska-Kucharska et al. (15) reported that age did not influence treatment efficacy, which is consistent with the results of our study.

While surgical treatment is preferred in younger patients with a nodule diameter greater than 3 cm, it has been reported that RAIT is also successful in the treatment of large nodules. Erdogan et al. (4) showed that the response to single-dose treatment was less effective in patients with larger nodule volume; however, even larger nodules were treated successfully with additional doses (2-5 times) administered at 6-month intervals. In our study, patients were evaluated according to the diameter of the largest nodule seen on USG and the cure rate was found to be higher in patients with nodule diameter ≤2 cm (100%) as compared to patients with nodule diameter >2 cm (92%), with the difference between the two groups being statistically significant.

In earlier studies, no statistically significant difference was found (7,13) between TMNG and TA in terms of treatment efficacy in TNG patients receiving RAIT. Knapska-Kucharska et al. (15) reported a cure rate of 75% for TMNG and 82% for TA (15). In the current study, which was consistent with the aforementioned studies, the cure rate was similar in both TA and TMNG patients, and there was no statistically significant difference (94.7% and 93.8%).

There is ongoing controversy on the use of ATD during RAIT. While many studies have reported that ATD use before or after RAIT reduces treatment success (16), others have stated that ATD do not affect treatment success (15,17). A study by Allahabadia et al. (5) reported that the ATD effect is seen at low doses, and that this can be overcome with the administration of higher doses. A meta-analysis of 14 studies reported that ATD use one week before or after treatment increased the risk of treatment failure, and reduced hypothyroidism rates (16). Another unresolved discussion is about how many days before the RAIT should ATD be discontinued. Some authors think that discontinuing ATD 2 days before treatment will increase efficacy by 50% through increased radioactive iodine retention and a longer effective half-life (18,19). Körber et al. (20) stated that ATD use did not affect RAIT results in Graves’ patients, but negatively affected treatment success in TNG patients. This has been interpreted as a possible “stealing phenomenon” in this patient group. Since TSH levels are higher during RAIT in TNG patients using ATD, this phenomenon may be caused by the TSH-stimulated normal thyroid tissue, therefore, preventing RAI uptake by toxic nodules. In the current study, the cure rate was similar in ATD users and ATD naive patients (93.8% vs. 94.6%), and hypothyroidism rate was relatively higher in ATD users as compared to others (39.2% vs. 28.2%). High doses were used in the current study and ATD use was discontinued 1 week prior to treatment and was restarted 1 week after treatment for possible exacerbation of hyperthyroidism. Since no adverse effects of ATD were observed and no patient developed thyroid storm in the current study, a one -week period can be considered to be sufficient for discontinuing ATD.

In conclusion, high cure rates were achieved with fixed I-131 dose of 555 MBq and 740 MBq in patients with TNG. Our results showed that nodule diameter and RAI dose are important factors for treatment success whereas age, gender, underlying etiology and antithyroid drugs do not have an impact on the outcome of RAIT.

## Figures and Tables

**Table 1:**
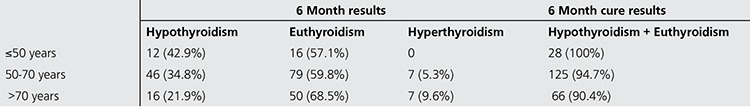
Treatment results according to age groups

**Table 2:**
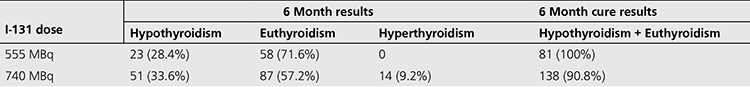
Treatment results according to the I-131 dose administered

**Table 3:**
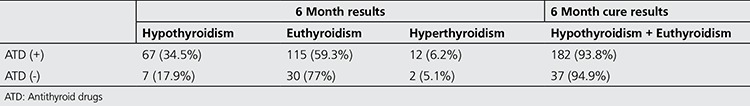
Treatment results according to the use of antithyroid drugs
